# Use of antibiotics for prevention and treatment of sinus lift infections: an umbrella review of systematic reviews and meta-analyses

**DOI:** 10.1186/s12903-025-07465-2

**Published:** 2025-12-18

**Authors:** Leonardo Díaz, Mariana Ivanković, Pablo Urrutia, Xavier Uriarte, Miguel Olivares, Alfredo Torres, Shengchi Fan, Eduardo Fernández

**Affiliations:** 1 Department of Prosthodontics, Faculty of Dentistry, University of Chile, Santiago, Chile; 2 Department of Stomatology, Faculty of Dentistry, Universidad de Sevilla, Seville, Spain; 3 Perioplastic Institute, Santiago, Chile; 4 Private Practice, Santiago, Chile; 5 Postgraduate Implant Dentistry Department, School of Dentistry, Universidad Andrés Bello, Santiago, Chile; 6 Private Practice, Puerto Varas, Chile; 7 Private Practice, Rancagua, Chile; 8 Laboratory of Experimental Immunology & Cancer, Faculty of Dentistry, University of Chile, Santiago, Chile; 9 Department of Conservative Dentistry, Faculty of Dentistry, University of Chile, Santiago, Chile; 10 Department of Oral and Maxillofacial Surgery, Plastic Surgery, University Medical Centre of the Johannes Gutenberg-University, Mainz, Germany; 11 Oral Surgery and Implantology, Faculty of Medicine and Health Sciences, University of Barcelona, Barcelona, Spain; 12 Department of Restorative Dentistry, Faculty of Dentistry, University of Chile, Santiago, Chile; 13 Instituto de Ciencias Biomédicas, Universidad Autónoma de Chile, Santiago, Chile

**Keywords:** Dental Implants, Maxillary Sinus, Sinus Floor Augmentation, Maxillary Sinusitis, Antibiotic Prophylaxis

## Abstract

**Background:**

Antibiotic use in sinus floor elevation (SFE) procedures remains controversial due to heterogeneous protocols and inconsistent clinical outcomes.

**Aim:**

To evaluate the effectiveness of systemic antibiotic regimens for the prevention and management of infections associated with SFE procedures, based on a synthesis of existing systematic reviews and meta-analyses.

**Methods:**

The study protocol was registered in the PROSPERO (CRD420251061400). Seven systematic reviews and meta-analyses (2008–2024) were included. Methodological quality was appraised using AMSTAR-2, overlap among primary studies was evaluated with the GROOVE tool, and certainty of evidence was assessed through the GRADE approach. A descriptive synthesis was performed, as methodological heterogeneity and overlap precluded meta-analytic pooling.

**Results:**

Infection rates following SFE ranged from 0.3% to 11.6%, with implant survival consistently above 90% regardless of antibiotic regimen. Prophylactic antibiotics were commonly prescribed (Amoxicillin or Amoxicillin/Clavulanate, 7–10 days), although no standardized protocol was identified. Reviews rated as high or moderate quality provided limited yet consistent evidence suggesting that antibiotics may reduce infection risk in high-risk situations (e.g., membrane perforation, extensive lateral approach, or systemic comorbidities). The overall certainty of evidence was low to very low.

**Conclusions:**

Current evidence suggests that antibiotic prophylaxis may be beneficial only in selected high-risk scenarios, whereas routine use appears unnecessary in uncomplicated SFE. Given the low certainty and heterogeneity of existing studies, recommendations should be interpreted cautiously, and future well-designed randomized trials are needed to define standardized antibiotic and non-antibiotic strategies for infection prevention.

**Supplementary Information:**

The online version contains supplementary material available at 10.1186/s12903-025-07465-2.

## Introduction

The maxillary sinus is a pyramid-shaped cavity within the maxillary bone, lined by a thin, specialized mucosa known as the Schneiderian or maxillary sinus membrane (SM), composed of pseudostratified ciliated columnar epithelium [1]. In contrast, the oral mucosa is characterized by a keratinized stratified squamous epithelium, adapted to mechanical stress and bacterial challenges [2, 3]. This histological distinction is significant, as it influences the local immune response, microbial colonization patterns, and ultimately, the susceptibility to infection and the choice of antibiotic (ATB) therapy [4].

Maxillary sinus floor elevation (SFE) procedures are widely utilized to enhance the vertical bone height in the posterior maxilla, facilitating successful dental implant placement in areas with insufficient native bone volume [5, 6]. The two primary techniques employed are the lateral window approach, first described by Tatum [7], and the transcrestal (osteotome) technique, introduced by Summers [8]. Additionally, various alternative minimally invasive methods have emerged, including osteotome-mediated SFE with osseodensification, balloon-assisted elevation, and hydraulic pressure techniques, all aimed at minimizing surgical trauma and improving patient comfort [9–12]. Although these procedures demonstrate high success rates, they are not without potential complications. The most frequently encountered complications include perforation of the SM and infection of the graft [13–15].

Infections following SFE are of particular concern due to their potential to result in graft failure, the development of chronic sinusitis, and frequently require surgical intervention [16, 17]. Clinical manifestations include localized swelling, purulent exudate, tenderness, and occasionally systemic symptoms such as fever [18]. Although reported incidence rates vary, reviews suggest a prevalence of infection between 1 and 12% after SFE procedures [14, 19], emphasizing the need for effective preventive strategies.

ATB prophylaxis has traditionally been employed to mitigate postoperative infections in implant dentistry. However, its routine use in SFE procedures remains controversial [20, 21]. The most frequently prescribed ATBs include Amoxicillin and Amoxicillin-clavulanic acid, owing to their efficacy against common oral and respiratory pathogens [21]. Nevertheless, variations in prescribed regimens—from single preoperative doses to extended postoperative courses—reflect a lack of consensus and standardized protocols [22–24]. Moreover, the rise of antimicrobial resistance poses additional challenges, urging a critical reassessment of indiscriminate ATB use in dental surgery [25]. Recent studies advocate for risk-based strategies, wherein ATB prophylaxis is designated for patients presenting systemic comorbidities, extensive SFEs, or experiencing intraoperative SM perforations [21, 25]. Complementary strategies, which include advanced surgical techniques, strict aseptic protocols, and thorough patient selection criteria, have shown similar effectiveness in reducing the occurrence of postoperative infections [22, 26].

Given the heterogeneity of clinical practices and the evolving nature of available recommendations, there remains an urgent need to consolidate and critically appraise current evidence. Although the 2022 Consensus Report of the Spanish Society of Implants (SEI- Sociedad Espanola de implantes) [22] provided valuable expert-based recommendations on preventive ATB therapy for implant procedures, it did not perform a meta-analytic or methodological evaluation of the available reviews, nor did it quantify evidence certainty or overlap. The present umbrella review (UR), complements that consensus by integrating all systematic reviews and meta-analyses (SR/MAs) published up to 2024 and assessing their methodological rigor (AMSTAR 2) [27], redundancy (GROOVE) [28], and certainty of evidence (GRADE) [29] to identify current gaps and strengthen evidence-based guidance for SFE surgery. Therefore, this UR aims to systematically analyze and synthesize findings from SR/MAs regarding the use of ATBs for the prevention and treatment of infections following SFE procedures, ultimately providing recommendations for evidence-based clinical decision-making.

## Materials and methods

### Protocol, registration, and study design

The protocol for this UR was prospectively registered in the PROSPERO database (registration ID: CRD420251061400). The scope, eligibility criteria, data extraction, and analytical approach were defined in advance to ensure methodological transparency and reproducibility.

This UR was conducted in accordance with the PRISMA 2020 (Preferred Reporting Items for Systematic Reviews and Meta-Analyses) statement [30] and cross-checked for compliance with the PRIOR 2022 (Preferred Reporting Items for Overviews of Reviews) guideline [31] and the methodological recommendations described in the Cochrane Handbook for SR of Interventions (Chapter V – Overviews of Reviews) [32]. Although there is currently no PRISMA extension developed exclusively for umbrella reviews, adherence to both PRISMA 2020 and PRIOR 2022 ensures comprehensive, transparent, and methodologically appropriate reporting consistent with current best practices for overviews of SRs.

### Focus question

The review was structured according to the PICO framework as follows:

- Population (P): Patients undergoing maxillary SFE procedures.
- Intervention (I): Use of prophylactic or therapeutic ATB regimens.
- Comparison (C): Placebo, no ATB, or alternative ATB regimens.
- Outcomes (O): The primary outcome was the effectiveness of ATB use, defined by objective clinical endpoints reported across the included SR/MA. These included: (1) postoperative infection or sinusitis incidence (%), (2) implant survival rate following SFE (%), (3) requirement for additional ATB therapy or surgical reintervention, and (4) clinical or radiographic resolution of infection. Secondary outcomes included adverse events related to ATB use and heterogeneity among regimens (type, timing, and duration).

All outcomes—both primary and secondary—were extracted and interpreted within the context of ATB use, comparing different prophylactic or therapeutic regimens (e.g., type, timing, and duration) or the absence of ATB administration, as reported in the included reviews.

### Eligibility criteria

#### Inclusion criteria

1. SR/MAs evaluating the use of systemic ATBs (prophylactic or therapeutic) in human patients undergoing maxillary SFE.
2. Reviews reporting outcomes related to the use of ATBs in the prevention and management of infections associated with SFE surgeries in terms of effectiveness, incidence of postoperative infections, complications related to infections, and implant survival rates. This criterion ensured inclusion of comprehensive sources providing infection-related data relevant to ATB use.
3. Articles published in English, Spanish, German, or Portuguese (to ensure comprehensive coverage of European and Latin American literature. No additional language filters were applied, and all databases were searched without restrictions to minimize potential language bias and maximize inclusivity).
4. There were no restrictions on the publication date.

#### Exclusion criteria

1. Narrative reviews, scoping reviews, conference abstracts, case reports, in vitro or animal studies.
2. Reviews that did not specifically assess infection-related outcomes in the context of SFE procedures.
3. SR without meta-analytic synthesis, unless they provide relevant quantitative outcomes.

### Information sources and search strategy

The literature search was performed independently by four reviewers (LD, MI, PU, and XU) in the Cochrane Library, PubMed, Ebsco, Scopus, and Web of Science. The algorithms used to search SR/MA were developed by an experienced reviewer (LD) and an experienced oral surgeon (MI), starting from the PubMed thesaurus (MeSH terms) that were adapted to the other platforms (Table S1).

### Study selection/screening

Studies were added to the Rayyan platform (https://www.rayyan.ai) to eliminate duplicates. They were screened by four reviewers (LD, MI, PU, and XU) by independent assessment of Title–abstract–keywords compared to the inclusion criteria. A Fleiss test was computed in Microsoft Excel 2022 (Microsoft Corporation, Redmond, USA) to assess inter-rater agreement for more than two reviewers. Interrater agreement was interpreted according to the categories proposed by Landis and Koch [33]. From this screening, studies compatible with full-text reading were independently selected. Discrepancies were resolved by discussion or consultation with an arbitrary reviewer (MO). The last search update was conducted up to May 5, 2025. In addition, manual search by Google Scholar and screening of the reference lists of eligible SR were performed to identify potentially relevant publications not captured through electronic searches.

### Data extraction and synthesis

Data extraction was performed by one reviewer (LD) and then checked by another (MI). Authors were contacted by e-mail to request the full texts of the articles in case they were unavailable or to obtain key information not reported in the included studies. A summary of study characteristics included (1) author(s) and year of publication, (2) focus, (3) number and type of primary studies included, (4) number of patients, (5) surgical approach, (6) ATB protocol, (7) complication rate, (8) implant survival rate, (9) follow-up period, (10) postoperative infection management, and (11) main findings.

A qualitative synthesis will be conducted. The primary outcome of interest is the reported incidence of infection following SFE procedures, and the relationship between ATB protocols and infection rates. Findings will be presented descriptively and summarized in structured tables. Subgroup findings (e.g., lateral vs. transcrestal approach) and the type of ATB used will also be discussed.

Given the substantial clinical and methodological heterogeneity across the included reviews—regarding populations (healthy vs. medically compromised), surgical approach (lateral vs. transcrestal), ATB agents, dosages, timing and duration, comparators (no ATB vs. alternative regimens), outcome definitions (infection/sinusitis, implant survival), and follow-up periods—we did not perform a de novo meta-analysis or re-pool estimates across reviews. Where individual reviews reported meta-analytic results, we present those pooled estimates verbatim, but we did not combine them further to avoid double-counting of primary studies and ecological bias arising from between-review differences. This approach follows Cochrane guidance for overviews of reviews [32], which recommends descriptive synthesis when heterogeneity and/or primary-study overlap. Consequently, results are reported as structured narrative summaries and ranges, with cross-review comparisons interpreted qualitatively.

### Certainty of evidence and overlap among primary studies

The certainty of the evidence reported in the included reviews was considered during interpretation. When available, we reported the original GRADE (Grading of Recommendations Assessment, Development and Evaluation) ratings provided by each review [29]. In cases where no formal GRADE assessment was performed, the overall strength of evidence was judged qualitatively based on consistency, directness, precision, and risk of bias. This differentiation between reported and author-assessed certainty was clearly maintained throughout the synthesis to ensure transparency and avoid overestimation of evidence strength.

To evaluate the degree of overlap among primary studies included in the selected SR, the GROOVE (Graphical Representation of Overlap for Overviews) tool was applied [28]. This approach allows visualization and quantification of overlap by constructing a citation matrix in which each row corresponds to a primary study and each column to an SR. The Corrected Covered Area (CCA) was then calculated to estimate the proportion of overlapping studies beyond what would be expected by chance [34]. The CCA, a validated index ranging from 0% (no overlap) to 100% (complete redundancy), was interpreted as follows: slight (0–5%), moderate (6–10%), high (11–15%), or very high (> 15%) overlap. The analysis was performed using a custom-built citation matrix in Microsoft Excel 2022 (Microsoft Corporation, Redmond, USA), following the methodology described by Pieper et al., 2014 [35], ensuring methodological reproducibility.

### Risk of bias assessment

The methodological quality and risk of bias of the included SR and meta-analyses were assessed using the AMSTAR 2 (A Measurement Tool to Assess Systematic Reviews 2) tool, a validated instrument specifically designed to evaluate the methodological rigor of SR that include both randomized and non-randomized studies of healthcare interventions [27]. Each included review was independently evaluated by two trained reviewers (LD, MI), and discrepancies were resolved through consensus or by consultation with a third reviewer (XU). Reviews were rated as high, moderate, low, or critically low quality, according to the guidance provided by the AMSTAR 2 developers, based on the presence or absence of critical and non-critical weaknesses. A high-quality rating was assigned when no or only one non-critical weakness was identified. Moderate quality was assigned when more than one non-critical weakness was present, but no critical flaws. Low-quality reviews exhibited one critical flaw, with or without non-critical weaknesses, while critically low-quality reviews had more than one critical flaw, severely limiting their reliability. In this UR, the AMSTAR 2 assessments were used to appraise the confidence in the findings and contextualize the evidence's strength when synthesizing data on ATB use for infection prevention and management in SFE procedures.

Although reviews with critically low or low methodological quality (according to AMSTAR-2) were retained for completeness, their influence on the synthesis was limited by weighting their findings qualitatively. Inferences and summary statements were primarily based on reviews rated as high or moderate quality, whereas results from lower-quality reviews were described but not used to support key conclusions. This approach follows current methodological guidance for umbrella reviews, emphasizing transparency while minimizing bias from heterogeneous evidence sources.

## Results

### Study selection

**Fig. 1:**
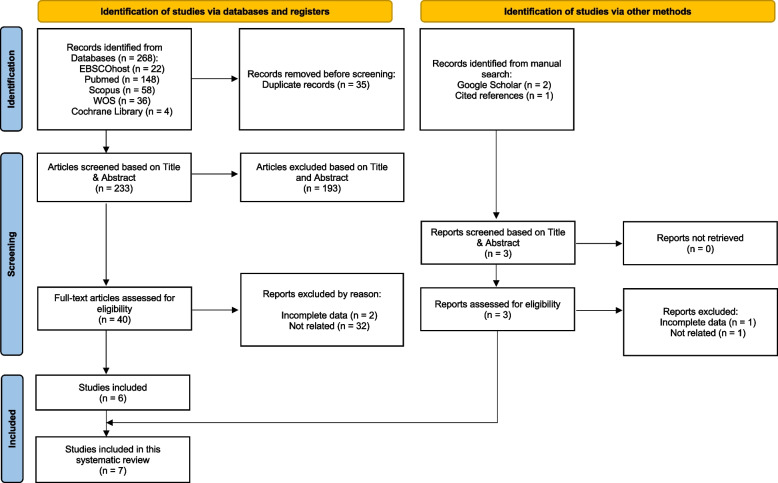
PRISMA flow diagram of the study selection process

A total of 268 records were initially identified through electronic database searches in PubMed, Cochrane Library, Scopus, Web of Science, and EBSCO. Additionally, three records were identified through Google Scholar and a manual search of reference lists. After merging and reviewing the results, 35 duplicate records were removed, leaving 236 articles for screening. Inter-reviewer agreement was high, with a Cohen’s kappa coefficient of 0.76, indicating substantial agreement. Discrepancies were resolved through discussion. Following Title-abstract-keywords screening, 43 full-text articles were retrieved for detailed evaluation. Of these, 7 SR and/or MA met the inclusion criteria and were included in the final synthesis. Agreement between reviewers for final inclusion was complete (100%). A total of 36 full-text articles were excluded, with reasons documented in Table S2. The study selection process is shown in Fig. 1.

### Characteristics of the SR/MAs included in the study

The seven included reviews corresponded to five SRs [14, 17, 21, 36, 37] and two more that included MA [38, 39], which were published between 2008 and 2024 and collectively encompassed 189 primary studies published from 1997 to 2022, including 80 retrospective studies, 46 prospective studies, 27 case series, 26 randomized clinical trials (RCTs), five cohort studies, four clinical trials, and two case–control studies. The total reported sample analyzed more than 18,900 patients with 28,000 implants placed in sinus-augmented areas. Of the included reviews, four focused on the prevention of sinus infection [21, 36, 37, 39], two on the management of infections [14, 38], and one addressed both aspects [17]. The surgical approaches assessed included both the lateral window and transcrestal SFE techniques.

A detailed summary of ATB regimens reported across the included reviews is presented in Tables 1 and 2. Considerable heterogeneity was observed in the choice of ATB, dosage, and duration. Amoxicillin and Amoxicillin/Clavulanate were the most commonly prescribed agents, often administered as single preoperative doses of 2 g orally 1 h before surgery or as extended postoperative regimens of 7–10 days. Clindamycin (300–600 mg every 6–8 h) was consistently recommended for patients with penicillin allergy. Some protocols combined ATBs with corticosteroids or nonsteroidal anti-inflammatory drugs (NSAIDs), while others included intravenous regimens such as ampicillin/sulbactam 3 × 3 g preoperatively followed by oral continuation.

**Table 1:** General characteristics of the systematic reviews and meta-analyses included in this study

**Study**	**Focus**	**Prevention/Management of Infection**	**Type of Study**	**Studies included and Sample Size**	**SFE** **Surgical Approach**	**Postoperative Infection** **Management**	**Main Findings**
Tan et al., 2008 [35]	Survival rate of implants placed in sites with transalveolar SFE	Prevention	SR	19 studies (9 PS, 10 RS) Patients: 2830 Implants: 4388 SFES: NR	Transalveolar: 4388 implants • Osteotome technique: 2831 implants • Hydraulic technique: 1557 implants	NR	High survival rates comparable to non-augmented sites. The transalveolar technique is predictable with a low incidence of complications.
Pjetursson et al., 2008 [36]	Survival rate of implants placed in sites with lateral approach SFE	Prevention	SR	48 studies (26 PS, 22 RS) Patients: 3716 Implants: 12020 SFES: NR	Lateral approach: 12020 implants	NR	High survival rates of implants placed in combination of SFES with lateral approach.
Salgado-Peralvo et al., 2023 [21]	Preventive Antibiotic Therapy in SFE	Prevention	SR	12 studies (1 RCT, 2 PS, 9 RS) Patients: 3534 Implants: 2063 (*reported*) SFES: 4178	Lateral approach: 4178 SFES	NR	Lack of consensus on prophylactic antibiotics for SFES.
Schiavo-Di Flaviano et al., 2024 [38]	Clinical Outcomes of Sinus Membrane Perforation	Prevention	SR/MA	10 studies (5 CHS, 2 CS, 1 CT, 2 CC) Patients: 1666 SFES: 2229 Implants: 5052	Lateral approach: SFES: 2229 Implants: 5052	Antibiotics NSAIDs CHX 0.12-0.2% Corticosteroids	Membrane perforation should not be considered a reason to abort the procedure or an absolute contraindication to implant placement.
Allevi et al., 2022 [37]	Management of Sinusitis following SFE	Management	SR/MA	8 studies (3 PS, 5 CS) Patients: 495 with dental implantation, 181 with sinusitis following dental implantation	NR	ESS for sinusitis treatment: 162/181 patients healed After ESS patients healed with: • Antibiotics: 7 patients • Sinus toilette: 1 patient • ESS (reintervention) + implant removal: 3 patients • ESS (reintervention) + OAF closure: 2 patients • Medical therapy: 5 patients • Lost of follow-up: 1 patient	ESS was the most frequent treatment of choice for sinusitis following dental implantation with high success rates.
Hsu et al., 2022 [14]	Complications in SFE	Management	SR	74 studies (20 CS, 25 RCT, 24 RS, 2 PS, 3 CT) Patients: 3419 Implants: 5236 *(reported)* SFES: 4411	Lateral approach: 3436 SFES Transcrestal approach: 975 SFES	Antibiotics Nasal decongestants	Transcrestal techniques with lower risk
Schlund et al., 2022 [17]	Management of Sinus Graft Infection	Prevention and Management	SR	18 studies (15 RS, 3PS) Patients: 3319 SFES: 1463 *(reported)*	NR	Antibiotics NSAIDs Nasal decongestants Implant Removal Medical Therapy	

*CC *Case Control, *CHS *Cohort Study, *CHX *Chlorhexidine, *CS *Case Series, *CT *Clinical Trial, *ESS *Endoscopic Sinus Surgery, *MA *Meta-Analysis, *NR *Not Reported, *OAF *Oro-antral Fistula, *PS *Prospective Study, *RCT *Randomized Controlled Trial, *RS *Retrospective Study, *SFE *Sinus Floor Elevation, *SFES *Sinus Floor Elevation Surgeries, *SR *Systematic Review

**Table 2:** Characteristics of Antibiotic regimen, dose, and posology of the included studies for Preventive Sinus Infection

**Study**	**Antibiotic regimen/number of implants**	**Antibiotics regimen/number of patients**	**Antibiotic regimen/number of SFES**	**Antibiotic dose and posology**
Tan et al., 2008 [36]	PreOp: 133 PreOp and PostOp: 1159 PostOp: 730 PostOp or none: 252 NR: 2114	PreOp: 74 PreOp and PostOp: 770 PostOp: 443 PostOp or none: 181 NR: 1362	NR	NR
Pjetursson et al., 2008 [36]	NR	PreOp: 76 PreOp and PostOp: 1930 PostOp: 427 None: 41 NR: 1242	NR	NR
Salgado-Peralvo et al., 2023 [21]	PreOp: 438 PostOp: 580	PreOp: 146 PreOp and PostOp: 2363 PostOp: 817 None: 123	PreOp: 237 PreOp and PostOp: 2507 PostOp: 1087	PreOp: 1) OA Amoxicillin/Clavulanate 875/125 mg/12 h, 1 d, starting 1 h PreOp. If SRPA: OA Clindamycin 300 mg/6 h, 1 d. (62 SFES) 2) OA Amoxicillin 3 g, 30 min PreOp. (18 Patients) 3) OA Ampicillin 2 g PreOp. If SRPA: OA Azithromycin (*unspecified posology)* (175 SFES) PreOp and PostOp: 1) OA Amoxicillin 2 g, 1h PreOp + 2 g per day, 10 d PostOp If SRPA, OA Clindamycin 1.2 g per day, 10 d PostOp (1874 SFES) 2) OA Ampicillin 2 g PreOp + Amoxicillin/Clavulanate 875/125 mg/12 h, 10 d PostOp. If SRPA: OA Clindamycin 600 mg PreOp + Azithromycin 250 mg/24 h, 10 d PostOp. (359 SFES) 3) IV Ampicillin/Sulbactam3×3g, 1 h PreOp + OA Ampicillin/Sulbactam 3 g × 374 mg, 6 d PostOp. (83 Patients) 4) OA Amoxicillin 2 g, 1h PreOp + Amoxicillin 500 mg/8 h, 7 d Post Op. If SRPA: OA Clindamycin 600 mg, 1h PreOp + 300 mg/6 h, 7 d PostOp. (274 SFES) PostOp: 1) OA Amoxicillin/Clavulanate 875/125 mg/12 h, 1 d, starting 1 PostOp. If SRPA: OA Clindamycin 300 mg/6 h, for 7 d PostOp. (175 SFES) 2) OA Amoxicillin 1 g/12 h, for 2 d* PostOp. *If the culture was negative, the PAT was discontinued and, if positive, an antibiotic adapted to the identified species was prescribed. (227 SFES) 3) OA Clindamycin 300 mg, 8 h, for 5 d PostOp. (151 SFES) 4) OA Sultamicillin 375 mg/8 h, 3 d PostOp. If SRPA: OA clindamycin 300 mg/8 h, 3 d PostOp. (340 SFES) 5) OA Ampicillin 250 mg/6h, 7 d PostOp. If SRPA: OA Clindamycin 150 mg/6h, 7 d Post Op. (100 SFES) 6) OA Amoxicillin per day, 6 d (*unspecified posology*). *92 SFES with topical sterile Metronidazole solution (25 mg). (94 SFES)
Schlund et al., 2022 [17]	NR	NR: 661 Pre and Post: 2276 Post: 382	NR: 374 Pre and Post: 600 Post: 489	PreOp and PostOp: 1) Amoxicillin (If SRPA: Clindamycin) PreOp + Amoxicillin/Clavulanate for 7 d + NSAIDs PostOp. (17 Patients) 2) Amoxicillin (If SRPA: Clindamycin) PreOp + Amoxicillin (If SRPA: Clindamycin) for 10 d PostOp. (1874 Patients) 3) Amoxicillin/Clavulanate PreOp + Amoxicillin/Clavulanate + Dexamethasone PostOp. (127 Patients) 4) Amoxicillin (If SRPA: Clindamycin) PreOp + Amoxicillin (If SRPA: Clindamycin) for 7 d + NSAIDs PostOp. (198 Patients) 5) Cephalosporin for 5 d+ Desamethasone PreOp + Cephalosporin + Desamethasone PostOp. (70 Patients) PostOp: 1) Amoxicillin for 7 d PostOp. (7 Patients) 2) Clindamycin for 5 d + NSAIDs PostOp. (116 Patients) 3) Antibiotic therapy for 5 d (*unspecified*) + NSAIDs PostOp. (259 patients) 4) Cephalosporin (*unspecified) *+ Desamethasone PostOp. (70 Patients)
Schiavo-Di Flaviano et al., 2024 [38]	NR: 691 Post: 4361	NR: 191 Post: 1475	NR: 248 Post: 1981	NR

*IV *Intravenous, *NR *Not reported, *NSAID *Non-steroidal anti-inflammatory drug, *OA *Oral administration, *PreOp *Preoperative, *PostOp *Postoperative, *SFES *Sinus Floor Elevation Surgeries, *SRPA *Self-reported penicillin allergy

The reported postoperative infection rates, complication frequencies, and implant survival outcomes were consistently analyzed in relation to the ATB regimen described in each review. These measures thus represent the clinical effectiveness of ATB prophylaxis or treatment, rather than general surgical outcomes. Reported infection rates ranged from 0.3% to 11.6%, highlighting the absence of consensus regarding optimal duration and dosage for prophylaxis in SFE. A comparison of postoperative complications and implant survival related to these regimens is summarized in Table 3.

**Table 3:** Characteristics of intraoperative and postoperative complications, implant survival, infection management success, and follow-up time

**Study**	**Intraoperative complications (%)**	**Post-operative infection (%)**	**Post-operative infection reasons**	**Infection management success**	**Implant survival rate or/with longest follow-up time**	**Mean follow-up time** **(range)**
Tan et al., 2008 [35]	3.8% Membrane perforation (0 - 21.4%)	0.8% (0 - 2.5%)	NR	NR	92.8% after 3 years	2.4 years (1 - 5 years)
Pjetursson et al., 2008 [36]	19.5% Membrane perforation (0 - 58.3%)	2.9% (0 - 7.4%)	NR	NR	90.1% after 3 years	2.8 years
Salgado-Peralvo et al., 2023 [21]	NR	(0 - 23.3%)	NR	NR	98.4% after 20.2 months	3-32 months
Allevi et al., 2022 [37]	NR	36.6% (11.8 - 100%)	SFE w/wo IP: 39.8% Peri-implantitis: 24.9% Implant displacement: 24.9% Dental implant placement: 10.5%	89.5% (after ESS) 94.7% (ESS + ATB/medical therapy/reintervention)	NR	NR
Hsu et al., 2022 [14]	LSFE: 17.75% Membrane perforation TSFE: 5.13% Membrane perforation	LSFE: 1.74% TSFE: 0.31%	NR	NR	91.0% after 132 months	(0 – 132 months)
Schlund et al., 2022 [17]	NR	0.5 – 11.6%	NR	100% (Disease-free sinus)	NR	NR
Schiavo-Di Flaviano et al., 2024 [38]	29.42% Membrane perforation (16.1 - 37%)	NR	NR	NR	97.7% after 25.15 months (implants placed without membrane perforation) 97.0% after 25.15 months (implants placed with membrane perforation)	25.15 months (6 – 98 months)

*NR *Not Reported, *ESS *Endoscopic Sinus Surgery, *LSFE *Lateral Sinus Floor Elevation, *IP *Implant Placement, *SFE *Sinus Floor Elevation, *TSFE *Transcrestal Sinus Floor Elevation w/wo: with or without

Subgroup findings derived from the included reviews revealed distinct trends across surgical and patient-related variables. The lateral window technique consistently showed a slightly higher rate of postoperative infection (1.7–2.9%) than the transcrestal approach (0.3–1.0%) (Tan et al., 2008 [36]; Pjetursson et al., 2008 [37]; Hsu et al., 2022 [14]). Despite these differences, implant survival remained comparable between both techniques, exceeding 90% after three years. With respect to graft materials, no review identified a statistically significant difference in infection rate among xenografts, allografts, or autogenous bone, though SM perforations were more frequent when particulate grafts were employed in lateral approaches (Schlund et al., 2022 [17]; Allevi et al., 2022 [38]).

Patient-related factors such as smoking, preexisting sinusitis, and SM perforation size were identified as independent modifiers of infection risk (Schiavo-Di Flaviano et al., 2024 [39]). The likelihood of infection increased notably when perforations exceeded 10 mm or when graft material displaced into the sinus cavity. These subgroup observations indicate that infection risk following SFE is influenced more by surgical integrity and patient susceptibility than by the specific ATB regimen used.

Due to the high clinical and methodological heterogeneity observed among the included reviews—including variability in ATB protocols, surgical techniques, and definitions of postoperative infection or implant failure—quantitative pooling of data was not appropriate. Therefore, infection and implant survival rates were reported as ranges derived from each review, providing a descriptive overview of the observed variability. This approach allows a transparent presentation of the available evidence without introducing bias through inappropriate statistical aggregation.

### Overlap of primary studies and certainty of evidence (GROOVE and GRADE analysis)

**Fig. 2:**
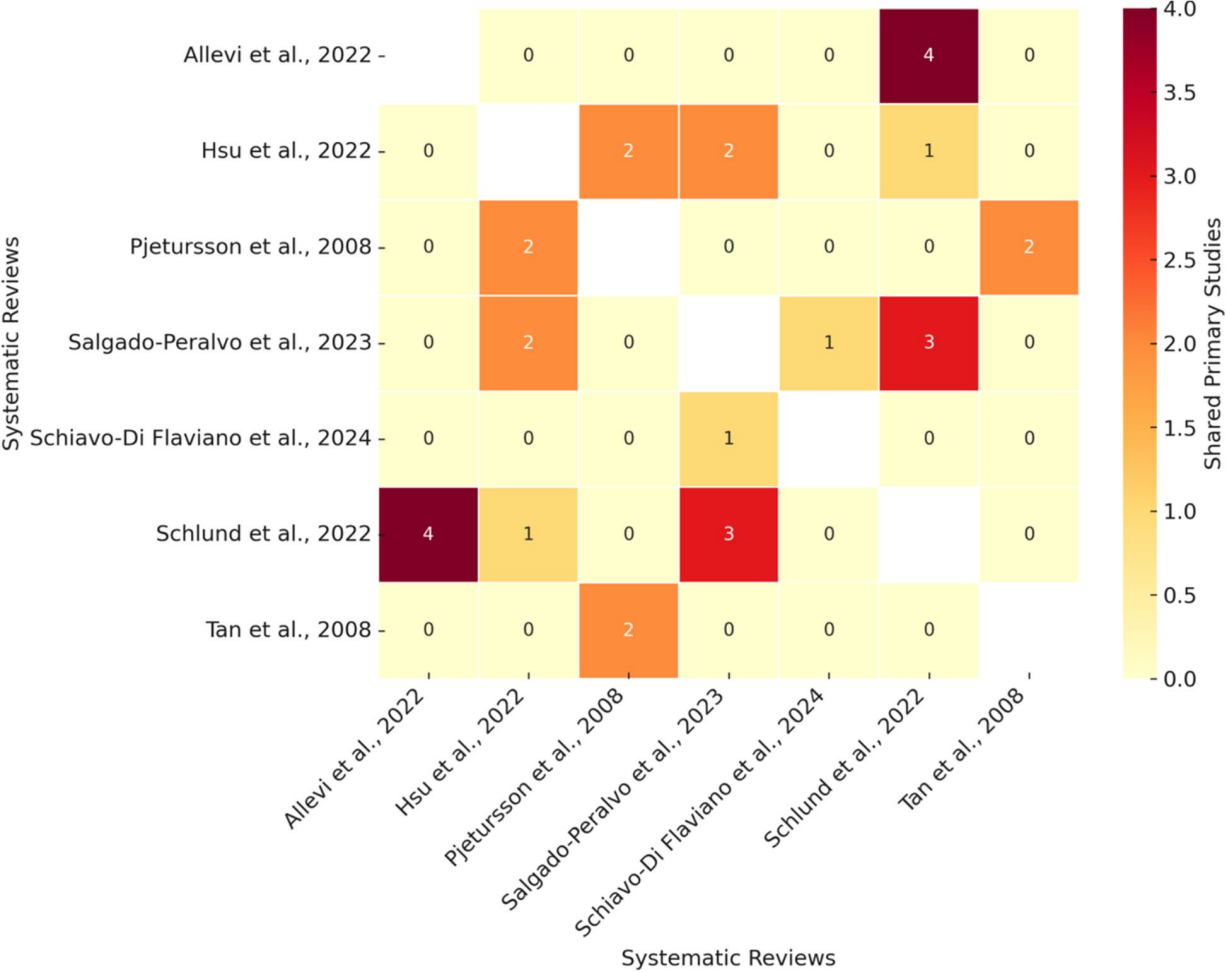
Groove matrix of shared primary studies between reviews. This heatmap displays the number of shared primary studies between pairs of systematic reviews included in the analysis. Each cell in the matrix represents the count of overlapping primary studies between two reviews, as indicated by the color intensity according to the scale bar on the right. Darker colors indicate a higher number of shared studies. The diagonal is left blank or excluded as it represents self-comparison. This visualization helps identify potential overlap or redundancy across systematic reviews on similar topics

The overlap analysis involved the construction of a citation matrix comprising 175 unique primary studies across the seven selected SR/MAs. Each study was coded for its presence or absence within each review, allowing the calculation of the CCA, a validated index used to estimate the proportion of overlap beyond chance [28, 34]. The calculated CCA was 1.33%, which falls within the category of slight overlap (0–5%), indicationg that, although some studies were shared among reviews—particularly between those addressing similar clinical questions, such as Tan et al., 2008 [36] and Pjetursson et al., 2008 [37]—most primary studies were unique to individual reviews. The overall low CCA supports the conclusion that the included SRs contribute largely independent bodies of evidence, enhancing the synthesized data's breadth and representativeness. Figure 2 graphically displays the number of shared primary studies between each pair of reviews, further confirming the minimal redundancy observed. These results indicate that while some degree of redundancy exists, it does not invalidate the overall synthesis but rather underscores the need for cautious interpretation of pooled evidence. As recommended by Pieper et al., 2014 [35], the CCA values and graphical representation were used to inform interpretation rather than as a measure of evidence strength. Consequently, the GROOVE analysis was applied to enhance transparency regarding study overlap rather than to quantify bias or weight specific reviews.

In parallel, the certainty of the evidence for each key clinical outcome was evaluated using the GRADE methodology [29]. This assessment considered several domains, including the nature of the primary study designs, risk of bias as determined by AMSTAR 2 ratings, inconsistency in results across studies, directness in addressing the clinical questions, and precision of effect estimates. For the primary outcome—namely, the effectiveness of ATB protocols in preventing postoperative infections following SFE procedures—the certainty of evidence was judged to be low. While a few included reviews incorporated RTCs, most of the underlying evidence stemmed from observational studies with variable methodological rigor. The risk of bias was moderate to high in several reviews, inconsistency was severe due to substantial heterogeneity in ATB type, timing, dosage, and outcome definitions, and imprecision was present in many cases due to limited sample sizes or wide confidence intervals. Nevertheless, the directness of the evidence was appropriate, as all studies focused specifically on SFE in dental implantology. Quantitative results of overlap are summarized in Table S3 and visualized in Fig. 2.

The certainty of outcomes related to the management of sinus graft infections was considered very low. This reflects the predominantly descriptive nature of the available evidence—often case series with incomplete reporting—and the absence of controlled comparisons. In contrast, the certainty of evidence regarding implant survival in patients receiving ATB prophylaxis versus those who did not was assessed as moderate.

These findings were generally supported by larger datasets and demonstrated more consistency in effect direction. Lastly, the comparative evaluation of different ATB regimens or dosing schedules yielded low-certainty evidence due to marked protocol heterogeneity and insufficient statistical power to detect differential effects.

Taken together, the GROOVE and GRADE assessments provide a dual perspective on the reliability of this UR. The minimal overlap in primary studies enhances confidence in the independence of the synthesized evidence base. At the same time, the GRADE evaluation underscores the need for cautious interpretation of findings and highlights areas where future high-quality, prospective research is warranted to strengthen the current evidence landscape.

### Risk of bias of included SRs (AMSTAR 2 assessment)

The methodological quality and potential risk of bias of the seven included SR/MAs were assessed using the AMSTAR 2 tool [27]. The evaluations revealed variable methodological quality across the studies. Two SRs were rated as high quality [21, 39], showing no critical flaws and only minor non-critical weaknesses. Both studies demonstrated rigorous design, protocol transparency, duplicate data handling, and robust reporting of included studies and risk of bias. Three reviews were rated as moderate quality [14, 36, 37]. These reviews showed strengths in literature search and synthesis, but were limited by omissions such as a lack of protocol registration, missing lists of excluded studies, or partial integration of risk of bias into their interpretation. One review [38] was rated as low quality, primarily due to the absence of a pre-registered protocol and inconsistent handling of bias across primary studies. The study lacked duplicate selection procedures and did not consistently report on funding sources or exclusion lists, which are critical for assessing transparency and reproducibility. The review by Schlund et al., 2022 [17] received a rating of critically low quality, as it failed to address several core AMSTAR 2 items, including protocol registration, risk of bias assessment, and the impact of methodological quality on the interpretation of findings. Additionally, the study did not perform study selection or data extraction in duplicate or provide a list of excluded studies, significantly limiting its reliability. These findings indicate that while most reviews presented moderate to high methodological standards, including lower-quality evidence, introduces limitations that must be considered when interpreting the synthesized conclusions of this UR A detailed breakdown of AMSTAR 2 item-level scores and global ratings is presented in Table 4.

**Table 4:** Critical Appraisal of Included Systematic Reviews According to AMSTAR 2

**AMSTAR 2 Item**	Tam et al., 2008 [35]	Pjetursson et al., 2008 [36]	Salgado-Peralvo et al., 2023 [21]	Schiavo-Di Flaviano et al., 2024 [38]	Allevi et al., 2022 [37]	Hsu et al., 2022 [14]	Schlund et al., 2022 [17]
1. Did the research questions and inclusion criteria for the review include the components of PICO?	N	N	Y	Y	N	N	N
2. Was the review protocol registered before commencement of the review (e.g., in PROSPERO)?	Y	Y	Y	Y	P	Y	P
3. Did the review authors explain their selection criteria clearly?	Y	Y	Y	Y	Y	Y	P
4. Did the review authors use a comprehensive literature search strategy?	Y	Y	Y	Y	N	Y	N
5. Was study selection performed in duplicate?	Y	Y	Y	Y	N	Y	N
6. Was data extraction performed in duplicate?	N	N	Y	Y	N	Y	N
7. Did the review authors provide a list of excluded studies and justify the exclusions?	Y	Y	Y	Y	P	Y	P
8. Were the characteristics of the included studies adequately described?	P	P	Y	Y	P	P	N
9. Did the review authors use a satisfactory technique for assessing risk of bias (RoB) in individual studies?	N	N	Y	P	N	N	N
10. Did the review authors use appropriate methods for statistical combination of results?	NA	NA	NA	Y	Y	NA	NA
11. Did the review authors account for RoB in individual studies when interpreting/discussing results?	N	N	Y	Y	N	P	N
12. Did the review authors assess the potential impact of heterogeneity?	Y	Y	Y	Y	P	Y	N
13. Did the review authors investigate publication bias (e.g., funnel plot, statistical tests)?	N	N	Y	Y	N	P	N
14. Did the review authors report any potential sources of conflict of interest, including funding received?	Y	Y	Y	Y	Y	Y	P
15. Did the review authors discuss the limitations of the review and/or included studies?	P	P	Y	Y	N	Y	N
16. Did the review authors assess the certainty/confidence in the body of evidence (e.g., GRADE)?	Y	Y	Y	Y	P	Y	P
**Overall quality**	**Moderate**	**Moderate**	**High**	**High**	**Low**	**Moderate**	**Critically Low**

*Y *Yes, *N *No, *P *Partial yes, *NA *Not Applicable

### Effectiveness of ATBs in the prevention and management of infections associated with SFE surgeries

The seven SRs and MA in this UR presented diverse but clinically significant findings addressing both prophylactic and therapeutic ATB strategies. Regarding infection prevention, three reviews focused primarily on evaluating prophylactic ATB regimens in patients undergoing SFE. Salgado-Peralvo et al., 2023 [21] specifically examined the effectiveness of preventive ATB therapy in SFE surgeries and identified a lack of standardization across studies in terms of ATB selection, dosage, timing, and duration. While most studies included in that review prescribed ATBs, the outcomes varied significantly, and the evidence did not demonstrate a clear superiority of any specific regimen over others. Nonetheless, the use of preoperative and combined perioperative regimens was frequently associated with a lower incidence of postoperative infection in comparison to postoperative-only protocols or the absence of ATBs. The earlier reviews by Tan et al., 2008 [36] and Pjetursson et al., 2008 [37], which evaluated implant survival and complication rates in transcrestal and lateral SFE techniques, respectively, provided indirect evidence supporting the role of ATBs in infection prevention. Although neither review was designed to test the effectiveness of ATB use specifically, both reported low postoperative infection rates, ranging from 0.8% in transcrestal approaches to 2.9% in lateral approaches, within cohorts that predominantly received perioperative ATB coverage.

Regarding infection management, three reviews [14, 17, 38] focused on the diagnosis, clinical presentation, and resolution of infections arising after SFE procedures, including graft infections and postoperative sinusitis. Allevi et al., 2022 [38] reported that surgical intervention via endoscopic sinus surgery (ESS) combined with systemic ATB therapy was the most effective management strategy, achieving clinical resolution in over 94% of cases. ATB monotherapy was less effective for more severe infections or chronic sinusitis when used in isolation. In addition, Schlund et al., 2022 [17] emphasized that early ATB therapy—often using Amoxicillin/Clavulanate, Clindamycin, or Fluoroquinolones—combined with adjunctive anti-inflammatory and decongestant therapy, was successful in most cases of graft infection, provided intervention occurred before extensive implant displacement or oro-antral fistula (OAF) formation. Finally, Hsu et al., 2022 [14], while focusing more broadly on complications associated with SFE, identified ATB therapy as a cornerstone of initial management for infection-related complications. However, their data also underlined that infections occurring in the context of SM perforation or extensive graft failure often required surgical intervention in addition to ATBs. Moreover, the review by Schiavo-Di Flaviano et al., 2024 [39], although primarily focused on membrane perforations, also reported high implant survival rates even in cases where perforations occurred, provided proper postoperative ATB prophylaxis and anti-inflammatory regimens were employed.

### Analysis of ATB regimens and adjunctive medical therapy

The complexity of ATB use in the context of SFE is further illustrated by the diversity of regimens reported across the included SRs. A detailed synthesis of ATB protocols employed across the included reviews is presented in Tables 2 and 5. In the reviews that examined prophylactic strategies for preventing postoperative infections, the most commonly prescribed ATBs included Amoxicillin, alone or combined with Clavulanic Acid. Clindamycin was frequently used for patients with a reported penicillin allergy. Additionally, Cephalosporins and Azithromycin were prescribed less often. The timing and duration of ATB administration varied substantially among studies. Some protocols employed single preoperative doses, while others implemented extended perioperative courses lasting up to 10 days postoperatively. The data indicate that the combination of preoperative and postoperative administration was the most frequently used protocol, although no consensus emerged regarding superiority. Furthermore, posology was inconsistently reported across studies, with several failing to specify exact dosing, route of administration, or duration.

**Table 5:** Characteristics of preoperative and postoperative medical treatment in relation to surgical treatment in the included studies of sinus infection management

**Study**	**Preoperative medical treatment**	**Postoperative medical treatment**
Allevi et al., 2022 [37]	• 500 mg Cefuroxime 2/d for 10 d + nasal steroid spray (Triamcinolone, 2 puffs per nostril 2/d for 1 week): 1 patient. • Topical nasal Oxymetazoline nasal HCl for 3 d + OA Moxifloxacin + OA Erdosteine + saline and nasal irrigation for 7 d (extended for another 7 d if clinical benefit): 19 patients. • OA Levofloxacin 500 mg 1/d for 5d before surgery (in case of allergy, Cefuroxime 500 mg 2/d for 5 d before surgery): 36 patients. • NR: 125 patients.	• Unespecified ATB for 7-10 d + CHX mouthwash + saline nasal irrigations + Mupirocin ointment: 20 patients. • Topical steroids + saline nasal irrigations over 4 weeks: 2 patients. • Unspecified ATB: 14 patients. • Amoxicillin/Clavulanate 1g 2/d or Levofloxacin 500 mg 1/d before and after surgery for overall 14 days + saline nasal sprays and nasal irrigations + undefined nasal unguent for 60-90 d after surgery + CHX mouthwash for 10-12 d: 16 patients. • OA Cefuroxime 500 mg 2/d for 6-7 d, or OA Levofloxacin 500 mg 1/d or OA Ciprofloxacin 500 2/d for 8-9 d + saline nasal irrigations + topical nasal gomenol oil for 30 d: 65 patients. • OA Levofloxacin 500 mg 1/d for 10 d (in case of allergy, Cefuroxime axetil 500 mg 2/d for 10 d) + saline nasal irrigations + topical nasal niaouli oil 2-3/d for 30 d: 36 patients • NR: 28 patients
Hsu et al., 2022 [14]	• NR	• OA Amoxicillin/Clavulanate + Fluticasone (nasal anti-inflammatory) + Gentamicin, mesna, fluocinolone, and budesonide 0.25 mg/mL (aerosol therapy) with sessions every 12 h for 1 week, followed by another week with daily sessions: *reported in 5 patients.* • OA Clindamycin 300 mg + Levofloxacin 500 mg every 12 h for 7 d: *reported in 2 patients.*
Schlund et al., 2022 [17]	• Antibiotic therapy (*unspecified*): 28 Patients • Implant removal: 17 Patients • Intraoral drainage: 1 Patient • Amoxicillin/Clavulanate for 7 d: 2 Patients • Amoxicillin/Clavulanate for 14 d: 2 Patients • Levofloxacin for 5 d: 128 Patients • Amoxicillin/Clavulanate for 8 d + NSAIDs + fluticasone + aerosol therapy (gentamicin, mesna, fluocinolone, budesonide) for 14 d: 127 Patients • Medical therapy: 257 Patients	• Amoxicillin/Clavulanate for 10 d + NSAIDs + nasal spray: 17 Patients • Amoxicillin/Clavulanate for 14 d (or Levoflaxacin) + NSAIDs: 7 Patients • Antibiotic therapy (*unspecified*): 452 Patients • Antibiotic therapy (*unspecified*) + NSAIDs: 116 Patients • Ciprofloxacin for 14 d + NSAIDs: 8 Patins • Levofloxacion for 10 d (or Cefuroxime Axetil): 128 Patients • Metronidazole for 7 d: 1874 Patients • Amoxicillin for 7 d: 7 Patients • Clindamycin for 10 d: 94 Patients • Cephalosporin therapy (cefazolin during hospitalization + Cefuroxime Axetil 6–7 d after discharge) OR Quinolone therapy (levofloxacin or ciprofloxacin during hospitalization + 8–9 d after discharge): 257 Patients • Ceftriaxone for 7-10 d: 20 Patients • Amoxicillin/Clavulanate for 7 d + NSAIDs + nasal decongestant: 198 Patients • Amoxicillin/Clavulanate or Cefazolin: 13 Patients

*ATB *Antibiotic, *CHX *Clorhexidine, *NR *Not Reported, *NSAID *non-steroidal anti-inflammatory drug, *OA *Oral administration, *W/wo *with or without

Regarding adjunctive medical therapies and ATB regimens used in the management of sinus infections following SFE, reported treatments included oral or intravenous ATBs, often in combination with nasal corticosteroids, topical antiseptics (e.g., Chlorhexidine), decongestants, and mucolytics. In more severe cases, ATB therapy was complemented by surgical interventions such as ESS or intraoral drainage. While Amoxicillin/Clavulanate and Levofloxacin were the most commonly used ATBs in treatment regimens, combining systemic antimicrobial therapy with adjunctive topical and anti-inflammatory agents appeared to be the most effective strategy for resolving infection without recurrence. The diversity in treatment combinations and lack of standardization underscore the need for more straightforward clinical guidelines based on high-quality comparative studies.

### Incidence of postoperative infections

Among the studies focusing on infection prevention, Tan et al., 2008 [36] reported a mean postoperative infection rate of 0.8% (range: 0–2.5%) in a total of 2,830 patients undergoing transcrestal SFE. Most patients received perioperative ATBs in this cohort, although regimens were not uniformly detailed. Pjetursson et al., 2008 [37] documented a mean infection rate of 2.9% (range: 0–7.4%) across 3,716 patients and 12,020 implants placed via lateral window approaches. Notably, this review also reported a SM perforation rate of 19.5%, suggesting that anatomical or procedural complications may contribute to elevated infection risk. In both reviews, infection was reported as a rare complication but carried significant implications for graft integration and implant survival. On the other hand, Salgado-Peralvo et al., 2023 [21] did not provide pooled infection rates but reported individual study values ranging from 0% to 23.3%, depending on the type of ATB regimen and duration. The wide range underscores the methodological variability and lack of consensus regarding ATB prophylaxis protocols. Despite this variability, the review emphasized that combined preoperative and postoperative regimens were more frequently associated with lower infection rates than postoperative-only strategies.

Reviews evaluating infection management reported higher incidence values, as expected due to their inclusion of infected cases. Allevi et al., 2022 [38] found that 36.6% of patients developed sinusitis following implant placement, with causes attributed to implant penetration into the sinus, peri-implantitis, and graft failure. Among these, the presence of infection secondary to SFE was documented in 39.8% of cases. Their review also reported that ESS combined with medical therapy resolved the infection in 94.7% of patients. In Hsu et al., 2022 [14], the incidence of infection was analyzed according to surgical technique. In lateral SFE cases, the infection rate was 1.74%, while in transcrestal SFE cases, the rate was substantially lower at 0.31%, consistent with the less invasive nature of the latter. These findings support the notion that SM integrity and the surgical access route are significant risk factors for infection. Furthermore, Hsu et al., 2022 [14] observed that membrane perforation occurred in 17.75% of lateral SFE procedures, potentially compounding infection risk. In their review of sinus graft infections, Schlund et al., 2022 [17], reported infection rates ranging from 0.5% to 11.6%, depending on the graft material, surgical approach, and management protocols. In many cases, infections were successfully treated with systemic ATBs and adjunctive therapy; however, in severe or refractory cases, implant removal and surgical debridement were necessary. Finally, Schiavo-Di Flaviano et al., 2024 [39], while not reporting direct infection rates, acknowledged the role of perforation in infection risk and noted that implant survival remained high even in the presence of membrane perforation when adequate postoperative antimicrobial therapy was administered.

In synthesis, postoperative infection rates following SFE procedures ranged from 0.3% to 11.6%, depending on the population analyzed and the presence of complications such as membrane perforation or implant displacement. The overall pooled interpretation suggests that with appropriate surgical technique, the incidence of postoperative infection remains low. However, when infections do occur, particularly in the presence of anatomical complications, they necessitate complex management protocols and may compromise clinical outcomes.

### Complications related to infections

Infectious complications following SFE procedures represent a significant clinical concern, with potential consequences that extend beyond localized discomfort to more severe outcomes such as graft loss, implant failure, and chronic sinus pathology. Across the seven SRs analyzed, various complications were identified, with frequencies and severities influenced by surgical technique, infection control strategies, and the presence of intraoperative incidents such as SM perforation. One of the most consistently reported complications was sinus graft failure, particularly when infection occurred early in the healing process. Schlund et al., 2022 [17] described multiple instances of graft degradation that necessitated the removal of the grafted material and, in several cases, explantation of the dental implant. Similarly, Allevi et al.,2022 [38] noted that sinusitis secondary to implant penetration or peri-implantitis often led to implant loss, especially when infection extended to adjacent structures or compromised the sinus ostium. In their review, implant removal was required in patients who developed persistent maxillary sinusitis unresponsive to medical management, often in combination with ESS and closure of OAF.

Additional complications related to infection included chronic sinusitis, mucosal thickening, and ostium obstruction, as highlighted by Hsu et al., 2022 [14]. These conditions were typically associated with symptomatic presentations such as purulent drainage, nasal congestion, and facial pain, requiring prolonged ATB therapy and, in more advanced cases, surgical debridement. OAF were also reported as sequelae of infected sites, particularly when necrosis or dehiscence of the SM occurred postoperatively. In such cases, secondary surgical intervention was necessary to achieve closure and prevent further microbial ingress. Although SM perforation is primarily a mechanical complication, its occurrence was frequently linked to increased infection risk, as discussed by Schiavo-Di Flaviano et al., 2024 [39]. While not inherently compromising implant survival, large or poorly managed perforations created favorable conditions for contamination. Other less frequent but clinically significant complications included implant displacement into the maxillary sinus and failure of osseointegration secondary to infection-induced inflammation. These findings underscore the importance of stringent infection control protocols and careful management of intraoperative complications, as infectious sequelae not only jeopardize implant and graft success but may also compromise long-term sinus health and require complex multidisciplinary interventions.

### Implant survival rates

Implant survival following SFE procedures was reported across all included SRs, with consistently high success rates observed despite variations in surgical technique, presence of infection, and use of ATBs. Tan et al., 2008 [36] reported a survival rate of 92.8% after a mean follow-up of 2.4 years for implants placed using the transcrestal approach, while Pjetursson et al., 2008 [37] observed a 90.1% survival rate over 2.8 years following the lateral window technique, despite a higher membrane perforation rate. More recent data from Schiavo-Di Flaviano et al., 2024 [39] indicated implant survival rates of 97.0% in cases with SM perforation and 97.7% when the membrane remained intact, suggesting that with appropriate ATB prophylaxis and postoperative care, perforation does not significantly compromise implant prognosis. Similarly, Salgado-Peralvo et al., 2023 [21] reported a pooled implant survival of 98.4% despite heterogeneity in ATB protocols and follow-up periods. Hsu et al., 2022 [14] documented a 91.0% survival rate over a maximum of 132 months, with slightly lower rates associated with infection-related complications or membrane perforation. In studies addressing infection management, Allevi et al., 2022 [38] and Schlund et al., 2022 [17] did not report comprehensive survival rates but emphasized that implant removal was necessary in a minority of infection cases, particularly when infections progressed to sinusitis or required surgical intervention. Collectively, these findings support the long-term viability of implants placed in sinus-augmented sites, even in the presence of infection or surgical complications, provided that appropriate ATB strategies and clinical management are employed.

## Discussion

The prevention and management of infectious complications following SFE represent critical challenges in contemporary implantology. This UR synthesized evidence from seven SR/MAs to assess the effectiveness of ATB regimens and their impact on clinical outcomes. While ATBs are widely employed for prophylactic and therapeutic purposes, the overall certainty of the evidence remains low to moderate, and clinical practices are marked by heterogeneity and inconsistency. The synthesis was intentionally performed descriptively, as the methodological and clinical heterogeneity across the included reviews precluded quantitative pooling. The SRs differed substantially in ATB type, dosage, timing, duration, surgical approach, and outcome definitions, which would have rendered any meta-analytic re-estimation unreliable. This decision aligns with Cochrane guidance for overviews of reviews [32], which recommends structured narrative synthesis when primary-study overlap or heterogeneity is substantial. Therefore, infection and implant survival rates were summarized as ranges, allowing a transparent representation of the available data without introducing statistical artifacts.

Prophylactic ATB administration is a common practice in SFE surgeries, aiming to mitigate the risk of postoperative infections. However, the evidence supporting this practice is inconclusive. A SR by Salgado-Peralvo et al., 2023 [21] found no statistically significant difference in implant failure rates between patients who received ATBs and those who did not, suggesting that routine ATB prophylaxis may not be universally necessary. Similarly, an RCT included in the same review reported no significant difference in sinus infection rates between different ATB regimens, further questioning the necessity of extended ATB courses [40]. In contrast, managing established sinus graft infections often necessitates a combination of surgical intervention and ATB therapy. Schlund et al., 2021 [17] conducted an SR encompassing 18 studies, highlighting the heterogeneity in treatment protocols for sinus graft infections. Despite the varied approaches, the review emphasized the importance of surgical debridement and ATB therapy to achieve disease-free sinuses. The same review noted that while all patients eventually achieved sinus health, graft and implant success outcomes were more variable, underscoring the complexity of managing such infections.

Beyond the variability of ATB regimens, several procedural and patient-related factors appear to modulate the risk of postoperative infection following SFE. The lateral window approach—although well established—showed slightly higher infection rates than the transcrestal technique, which may be attributed to the greater surgical exposure and membrane manipulation rather than ATB prophylaxis per se (Tan et al., 2008 [36]; Pjetursson et al., 2008 [37]; Hsu et al., 2022 [14]). In contrast, the transcrestal approach, being less invasive, was consistently associated with lower infection incidence while maintaining comparable implant survival rates.

It should also be noted that some of the included SRs, such as those by Tan et al., 2008 [36], Pjetursson et al., 2008 [37], and Hsu et al., 2022 [14], reported postoperative infection outcomes and ATB protocols as secondary findings rather than primary objectives. Their inclusion was nevertheless justified, as these reviews provide fundamental quantitative data on infection rates, ATB use, and perioperative management within SFE procedures. Incorporating such reviews allows for a more comprehensive understanding of infection control and prophylaxis patterns, complementing the findings of more recent reviews that focused primarily on ATB efficacy.

With regard to graft materials, no consistent differences in infection rate were identified among xenogeneic, allogeneic, or autogenous grafts, yet membrane perforation occurred more frequently when particulate materials were used with lateral window techniques (Schlund et al., 2022 [17]; Allevi et al., 2022 [38]). Moreover, patient-specific factors—particularly smoking, pre-existing sinus pathology, and the presence of large membrane perforations—were recurrently reported as key modifiers of infection risk (Schiavo-Di Flaviano et al., 2024 [39]). These findings suggest that postoperative infection outcomes depend primarily on surgical integrity and patient susceptibility, while the contribution of ATBs remains secondary. Therefore, ATB prophylaxis should be considered an adjunct to, rather than a substitute for, meticulous surgical technique, proper case selection, and perioperative management tailored to the individual patient’s risk profile.

From a methodological perspective, the overall certainty of evidence rated through the GRADE approach was predominantly low to very low for outcomes related to both infection prevention and management. This limitation arises mainly from the heterogeneity in study design, small sample sizes, and the absence of randomized comparisons between ATB protocols. Consequently, the conclusions of this UR should be interpreted with caution. Although most SRs reported favorable outcomes associated with ATB prophylaxis, the low certainty reduces the confidence in these findings and precludes strong clinical recommendations.

In clinical practice, this implies that the decision to prescribe ATB after SFE should be individualized, considering patient comorbidities, surgical complexity, and the risk of SM perforation. The findings of this review therefore support a risk-based and evidence-informed approach, in line with current antimicrobial stewardship strategies rather than routine ATB use.

Anatomical factors, such as the presence of sinus septa and the thickness of the SM, play a crucial role in the risk of membrane perforation during SFE procedures. Membrane perforation is a common intraoperative complication, with reported incidence rates in the literature from 10 to 35% [41], values similar to those found in this UR, which highlights the wide range reported by Pjetursson et al., 2008 [37], from 0 to 58.3%. Multiple SM perforation repair protocols have been described [19, 42, 43], while small perforations can often be managed successfully with collagen membranes or platelet-rich fibrin, larger perforations may increase the risk of postoperative infections and graft failure.

Microbiologically, Streptococcus pneumoniae, Haemophilus influenzae, and Moraxella catarrhalis are the most commonly identified organisms in post-surgical sinusitis, with resistance to beta-lactam ATBs becoming increasingly reported [24, 44]. Consequently, while Amoxicillin/Clavulanate remains the first-line empirical treatment in most protocols, failure to respond within the first 7–10 days may warrant a switch to second-line agents such as Moxifloxacin, particularly in regions with high resistance prevalence [23, 24, 45]. However, the emergence of ATB-resistant pathogens necessitates cautious use of broad-spectrum ATBs. Antimicrobial stewardship principles advocate for the judicious use of ATBs to minimize the development of resistance [22, 46]. These considerations underscore the importance of interdisciplinary coordination between implant surgeons and otolaryngologists, which has been shown to improve diagnostic accuracy, expedite the resolution of infectious complications, and preserve long-term functional and esthetic outcomes in sinus-augmented implant rehabilitations [23, 47, 48]. Despite these challenges, implant survival rates following SFE procedures remain high. Tan et al., 2008 [36] reported an implant survival rate of 98.3% after three years when using rough-surfaced implants with membrane coverage of the lateral window. Similarly, Pjetursson et al., 2008 [37] found high survival rates with the transalveolar technique, although the evidence was deemed to have a high risk of bias. These findings suggest that successful implant outcomes are achievable with appropriate surgical techniques and postoperative care, even with complications.

The widespread use of broad-spectrum ATBs, particularly Amoxicillin and Amoxicillin/Clavulanate, raises growing concerns regarding antimicrobial resistance. Although these agents remain the most prescribed for prophylaxis in SFE, their indiscriminate or prolonged use contributes to the selection of resistant oral and respiratory flora. Current evidence underscores the need for antimicrobial stewardship in oral surgery, advocating for ATB prescription only when clinically justified by patient risk or intraoperative complications [26]. In this context, targeted prophylaxis—restricted to high-risk cases such as extensive SFEs, membrane perforations, or patients with systemic comorbidities—should be favored over routine coverage. Furthermore, several non-ATB preventive strategies have demonstrated comparable efficacy, including meticulous surgical technique, strict asepsis, preoperative chlorhexidine rinses, and the use of minimally invasive approaches. These measures align with global efforts to reduce unnecessary ATB exposure while maintaining patient safety in implant-related procedures.

The interpretation of findings in this umbrella review was weighted according to the methodological quality of the included SRs. Greater emphasis was given to results derived from reviews rated as high or moderate quality by AMSTAR-2 [27], while findings from critically low or low-quality reviews were described but not used to support key conclusions. This weighting approach ensured that the synthesis remained transparent and evidence-based despite heterogeneity across studies. Moreover, the overall certainty of evidence, as assessed through GRADE [29], was predominantly low to very low, emphasizing that the conclusions presented should be interpreted with caution. These considerations reinforce that current recommendations on ATB prophylaxis and treatment following SFE should be regarded as suggestive rather than definitive, pending confirmation by future high-quality randomized trials.

This UR presents several limitations that must be considered when interpreting the results. The included SR/MAs varied widely in methodological quality, as reflected by AMSTAR 2 ratings, with only a few classified as high or moderate quality. Heterogeneity among primary studies—regarding ATB regimen, surgical approach, and reporting standards—further limited comparability. Inconsistencies in outcome definitions (infection, implant survival, or postoperative complications) and follow-up durations also restricted the ability to perform quantitative synthesis. Moreover, the substantial heterogeneity among the included SRs —stemming from differences in primary study design, ATB regimen reporting, and outcome definitions—represents a critical limitation of this UR. Many reviews combined data from both randomized and observational studies, which differ considerably in internal validity. Infection outcomes were inconsistently defined, ranging from clinical diagnosis to radiographic or patient-reported parameters, and follow-up periods varied widely. These factors precluded meaningful quantitative synthesis and limited the capacity to establish causal inferences regarding ATB efficacy.

This methodological variability underscores the urgent need for standardized research protocols in future investigations. Uniform diagnostic criteria for postoperative sinus infection, consistent reporting of ATB dosage, timing, and duration, and harmonized outcome measures (e.g., infection rate, implant survival, and graft success) are essential to enable valid comparisons and meta-analytic aggregation. Establishing such standards will enhance reproducibility, improve evidence certainty, and ultimately support the development of clinical guidelines based on robust data. Although the search strategy included studies in English, Spanish, German, and Portuguese, potentially relevant reviews in other languages might not have been captured. Nevertheless, this minor limitation is unlikely to have influenced the conclusions due to the broad linguistic coverage of the databases searched.

Future research should focus on well-designed RCTs specifically tailored to the clinical scenarios most relevant to SFE procedures. These studies should clearly define and stratify patient populations—distinguishing healthy individuals from those with systemic comorbidities such as diabetes, smoking, or chronic sinus pathology—and differentiate between lateral and transcrestal surgical approaches. Key outcomes to be measured should include standardized definitions of postoperative infection (clinical and radiographic), graft integrity, membrane perforation healing, implant survival, and long-term sinus function. In addition, microbiological and antimicrobial resistance profiles of sinus flora should be investigated to support evidence-based ATB selection. Comparative RCTs assessing short versus extended ATB courses, ATB versus placebo, and systemic versus local antimicrobial strategies would be particularly informative. Furthermore, the integration of patient-reported outcomes—such as postoperative pain, swelling, and quality of life—would provide a more comprehensive assessment of clinical efficacy and patient-centered care. Finally, multicenter trials using standardized protocols and consistent follow-up periods are essential to reduce heterogeneity and to facilitate meaningful meta-analytic synthesis in future umbrella reviews.

## Conclusions

This UR synthesized and critically appraised the available evidence on the use of antibiotics for the prevention and management of infections associated with SFE procedures. The current evidence suggests that ATB prophylaxis may reduce postoperative infection risk in selected high-risk situations—such as SM perforation, extensive lateral approaches, or in patients with systemic comorbidities (e.g., diabetes, smoking, or chronic sinusitis). However, the overall certainty of evidence remains low to very low, and there is no consensus regarding the optimal antibiotic type, dosage, or duration.

In uncomplicated SFE cases or transcrestal approaches performed under strict aseptic conditions, routine antibiotic use appears unnecessary, as infection rates are low even without prophylaxis. Non-ATB preventive strategies—including meticulous surgical technique, antiseptic rinses, and the selection of biomaterials with antibacterial or low-contamination potential—represent viable adjuncts for infection control while supporting antimicrobial stewardship.

These conclusions should therefore be interpreted with caution, as they derive from heterogeneous and predominantly low-certainty data. Further well-designed randomized clinical trials and high-quality systematic reviews are required to establish standardized antibiotic and non-antibiotic protocols for sinus floor elevation procedures.

## Supplementary Information

Supplementary Material 1: Table S1: Search queries. Table S2: Articles excluded by reasons. Table S3: Summary of Certainty of Evidence (GRADE) for Key Outcomes Related to Antibiotic Use in SFE Procedures.

## Data Availability

The data sets used and/or analyzed during the current study are available from the corresponding author on reasonable request.

## Abbreviations

AMSTAR 2: A Measurement Tool to Assess Systematic Reviews 2
AZ: Azimuth Zone
ATB: Antibiotic
CC: Case Control
CCA: Corrected Covered Area
CHS: Cohort Study
CHX: Chlorhexidine
CS: Case Series
CT: Clinical Trial
ESS: Endoscopic Sinus Surgery
GRADE: Grading of Recommendations Assessment, Development and Evaluation
GROOVE: Graphical Representation of Overlap for OVErviews
IP: Implant Placement
LSFE: Lateral Sinus Floor Elevation
MA: Meta-Analysis
NR: Not Reported
NSAID: Non-Steroidal Anti-Inflammatory Drug
OA: Oral Administration
OAF: Oro-antral Fistula
PICO: Population, Intervention, Comparison, Outcome
PostOp: Postoperative
PreOp: Preoperative
PS: Prospective Study
RCT: Randomized Controlled Trial
RoB: Risk of Bias
RS: Retrospective Study
SFE: Sinus Floor Elevation
SM: Sinus Membrane
SR: Systematic Review
SR/MA: Systematic Review and/or Meta-Analysis
SRPA: Self-Reported Penicillin Allergy
UR: Umbrella Review
