# Supplementary material for: Use of antibiotics for prevention and treatment of sinus lift infections: an umbrella review of systematic reviews and meta-analyses
Source: BMC Oral Health. 2025 Dec 18;26:152. doi: 10.1186/s12903-025-07465-2 (PMC12829212; doi:10.1186/s12903-025-07465-2)
Supplement: Supplementary file 1 — Supplementary Material 1: Table S1: Search queries. Table S2: Articles excluded by reasons. Table S3: Summary of Certainty of Evidence (GRADE) for Key Outcomes Related to Antibiotic Use in SFE Procedures. [file 12903_2025_7465_MOESM1_ESM.docx]

**Supplementary File 1**

**Table S1.** Search queries

| **Database** | **Query** | **Filters** | **Results** |
| --- | --- | --- | --- |
| PubMed | ("Humans"[Mesh] OR “Dental Implantation, Endosseous”[Mesh] OR “Dental Implants”[Mesh]) AND (“Maxillary Sinus”[Mesh] OR “Maxillary Sinusitis”[Mesh] OR “Sinus Floor Augmentation”[Mesh] OR “Sinusitis”[Mesh] OR “Sinus graft” OR “Sinus Lift” OR “Sinus grafting” OR “Sinus Elevation” OR “Paranasal sinus” OR “Rhinosinusitis” OR “Sinus floor lift”) AND (“Postoperative Complications”[Mesh] OR “Anti-Bacterial Agents”[Mesh] OR “Antibiotic Prophylaxis”[Mesh] OR “Antibiotics” OR “prophylaxis” OR “Antibiotherapy”) | Article type:   1. Systematic Review 2. Meta-Analysis   Article Language:   1. English 2. German 3. Portuguese 4. Spanish | 148 |
| Web of Science | (ALL=(“Humans”) OR ALL=(“Dental Implantation, Endosseous”) OR ALL=(“Dental Implants”)) AND (ALL=(“Maxillary Sinus”) OR ALL=(“Maxillary Sinusitis”) OR ALL=(“Sinus Floor Augmentation”) OR ALL=(“Sinusitis”) OR ALL=(“Sinus graft”) OR ALL=(“Sinus Lift”) OR ALL=(“Sinus grafting”) OR ALL=(“Sinus elevation”) OR ALL=(“Paranasal sinus”) OR ALL=(“Rhinosinusitis”) OR ALL=(“Sinus floor lift”)) AND (ALL=(“Postoperative Complications”) OR ALL=(“Anti-Bacterial Agents”) OR ALL=(“Antibiotic Prophylaxis”) OR ALL=(“Antibiotics”) OR ALL=(“prophylaxis”) OR ALL=(“Antibiotherapy”)) | Document type:   1. Review Article   Article Language:   1. English | 36 |
| Scopus | ( TITLE-ABS-KEY ( "Dental Implantation, Endosseous" ) OR TITLE-ABS-KEY ( "Dental Implants" ) ) AND ( TITLE-ABS-KEY ( "Maxillary Sinus" ) OR TITLE-ABS-KEY ( "Maxillary Sinusitis" ) OR TITLE-ABS-KEY ( "Sinus Floor Augmentation" ) OR TITLE-ABS-KEY ( "Sinusitis" ) OR TITLE-ABS-KEY ( "Sinus graft" ) OR TITLE-ABS-KEY ( "Sinus Lift" ) OR TITLE-ABS-KEY ( "Sinus grafting" ) OR TITLE-ABS-KEY ( "Sinus elevation" ) OR TITLE-ABS-KEY ( "Paranasal sinus" ) OR TITLE-ABS-KEY ( "Rhinosinusitis" ) OR TITLE-ABS-KEY ( "Sinus floor lift" ) ) AND ( TITLE-ABS-KEY ( "Postoperative Complications" ) OR TITLE-ABS-KEY ( "Anti-Bacterial Agents" ) OR TITLE-ABS-KEY ( "Antibiotic Prophylaxis" ) OR TITLE-ABS-KEY ( "Antibiotics" ) OR TITLE-ABS-KEY ( "prophylaxis" ) OR TITLE-ABS-KEY ( "Antibiotherapy" ) ) AND ( LIMIT-TO ( DOCTYPE , "re" ) ) | Document type:   1. Review Article   Article Language:   1. English 2. German | 58 |
| Cochrane Library | ("Dental Implants" OR "Dental Implantation, Endosseous") AND ("Maxillary Sinus" OR "Maxillary Sinusitis" OR "Sinus Floor Augmentation" OR "Sinusitis" OR "Sinus graft" OR "Sinus Lift" OR "Sinus grafting" OR "Sinus Elevation" OR "Paranasal sinus" OR "Rhinosinusitis" OR "Sinus floor lift") AND ("Postoperative Complications" OR "Antibiotics" OR "Antibiotic Prophylaxis" OR "prophylaxis" OR "Antibiotherapy") | Document type:   1. Review Article | 4 |
| EBSCOhost | (("Dental Implants" OR "Dental Implantation, Endosseous")) AND (("Maxillary Sinus" OR "Maxillary Sinusitis" OR "Sinus Floor Augmentation" OR "Sinusitis" OR "Sinus graft" OR "Sinus Lift" OR "Sinus grafting" OR "Sinus Elevation" OR "Paranasal sinus" OR "Rhinosinusitis" OR "Sinus floor lift")) AND (("Postoperative Complications" OR "Antibiotics" OR "Antibiotic Prophylaxis" OR "prophylaxis" OR "Antibiotherapy")) AND (("systematic reviews" OR "meta analysis" OR "meta-analysis")) | Article Language:   1. English 2. Spanish | 22 |
| **TOTAL** | | | **268** |

**Table S2.** Articles excluded by reasons

| Esposito et al., 2009(1) | Insufficient data |
| --- | --- |
| Kim et al., 2019(2) | Insufficient data |
| Jeong et al., 2016(3) | Not related |
| Calin et al., 2014(4) | Not related |
| Stacchi et al., 2020(5) | Not related |
| Lee et al., 2023(6) | Insufficient data |
| Soares et al., 2024(7) | Insufficient data |
| Kämmerer et al., 2023(8) | Not related |
| Saltagi et al., 2021(9) | Not related |
| Huang & Zhou et al., 2019(10) | Not related |
| Lemiengre et al., 2018(11) | Not related |
| Dongo et al., 2018(12) | Not related |
| Cruz et al., 2018(13) | Not related |
| Geminiani et al., 2017(14) | Not related |
| Patel et al., 2017(15) | Not related |
| Burgstaller et al., 2016(16) | Not related |
| Head et al., 2016(17) | Not related |
| Thoma et al., 2015(18) | Not related |
| Rudmik & Soler, 2015(19) | Not related |
| Atieh et al., 2015(20) | Not related |
| Ahovuo-Saloranta et al., 2014(21) | Not related |
| Lemiengre et al., 2012(22) | Not related |
| Guarch Ibáñez et al., 2011(23) | Not related |
| Falagas et al., 2009(24) | Not related |
| Young et al., 2008(25) | Not related |
| Falagas et al., 2008(26) | Not related |
| Karageorgopoulos et al., 2008(27) | Not related |
| Ioannidis et al., 2001(28) | Not related |
| Benninger et al., 2000(29) | Not related |
| De Ferranti et al., 1998(30) | Not related |
| De Bock et al., 1997(31) | Not related |
| Grunau & Terheyden, 2023(32) | Not related |
| Striezel, 2004(33) | Not related |
| Jamali et al., 2020(34) | Insufficient data |
| Duttenhoefer et al., 2013(35) | Not related |

**References**

1. Esposito M, Grusovin MG, Felice P, Karatzopoulos G, Worthington H V, Coulthard P. Interventions for replacing missing teeth: horizontal and vertical bone augmentation techniques for dental implant treatment. Cochrane Database of Systematic Reviews. 2009 Oct 7;

2. Kim JS, Choi SM, Yoon JH, Lee EJ, Yoon J, Kwon SH, et al. What Affects Postoperative Sinusitis and Implant Failure after Dental Implant: A Meta‐analysis. Otolaryngology–Head and Neck Surgery. 2019 Jun 19;160(6):974–84.

3. Jeong KI, Kim SG, Oh JS, You JS. Implants Displaced Into the Maxillary Sinus. Implant Dent. 2016 Aug;25(4):547–51.

4. Călin C, Petre A, Drafta S. Osteotome-Mediated Sinus Floor Elevation: A Systematic Review and Meta-Analysis. Int J Oral Maxillofac Implants. 2014 May;29(3):558–76.

5. Stacchi C, Troiano G, Berton F, Lombardi T, Rapani A, Englaro A, et al. Piezoelectric bone surgery for lateral sinus floor elevation compared with conventional rotary instruments: A systematic review, meta-analysis and trial sequential analysis. Int J Oral Implantol (Berl). 2020;13(2):109–21.

6. Lee CT, Choksi K, Shih MC, Rosen P, Ninneman S, Hsu YT. The Impact of Sinus Floor Elevation Techniques on Sinus Membrane Perforation: A Systematic Review and Network Meta-analysis. Int J Oral Maxillofac Implants. 2023 Jul;38(4):681–96.

7. Soares L, Malzoni C, Silveira M, Junior E, Pigossi S. Evaluation of Different Approaches for Sinus Membrane Perforation Repair During Sinus Elevation: A Systematic Review and Meta-analysis. Int J Oral Maxillofac Implants. 2024 Feb 27;

8. Kämmerer PW, Fan S, Aparicio C, Bedrossian E, Davó R, Morton D, et al. Evaluation of surgical techniques in survival rate and complications of zygomatic implants for the rehabilitation of the atrophic edentulous maxilla: a systematic review. Int J Implant Dent. 2023 May 17;9(1):11.

9. Saltagi MZ, Comer BT, Hughes S, Ting JY, Higgins TS. Management of Recurrent Acute Rhinosinusitis: A Systematic Review. Am J Rhinol Allergy. 2021 Nov 23;35(6):902–9.

10. Huang Z, Zhou B. Clarithromycin for the treatment of adult chronic rhinosinusitis: a systematic review and meta‐analysis. Int Forum Allergy Rhinol. 2019 May 10;9(5):545–55.

11. Lemiengre MB, van Driel ML, Merenstein D, Liira H, Mäkelä M, De Sutter AI. Antibiotics for acute rhinosinusitis in adults. Cochrane Database of Systematic Reviews. 2018 Sep 10;2018(9).

12. Dongo V, von Krockow N, Martins-Filho PRS, Weigl P. Lateral sinus floor elevation without grafting materials. Individual- and aggregate-data meta-analysis. Journal of Cranio-Maxillofacial Surgery. 2018 Sep;46(9):1616–24.

13. Cruz RS, Lemos CA de A, Batista VE de S, Oliveira HFF e, Gomes JM de L, Pellizzer EP, et al. Short implants versus longer implants with maxillary sinus lift. A systematic review and meta-analysis. Braz Oral Res. 2018 Sep 13;32(0).

14. Geminiani A, Tsigarida A, Chochlidakis K, Papaspyridakos P V, Feng C, Ercoli C. A meta-analysis of complications during sinus augmentation procedure. Quintessence Int. 2017;48(3):231–40.

15. Patel ZM, Thamboo A, Rudmik L, Nayak J V, Smith TL, Hwang PH. Surgical therapy vs continued medical therapy for medically refractory chronic rhinosinusitis: a systematic review and meta-analysis. Int Forum Allergy Rhinol. 2017 Feb;7(2):119–27.

16. Burgstaller JM, Steurer J, Holzmann D, Geiges G, Soyka MB. Antibiotic efficacy in patients with a moderate probability of acute rhinosinusitis: a systematic review. Eur Arch Otorhinolaryngol. 2016 May;273(5):1067–77.

17. Head K, Chong LY, Piromchai P, Hopkins C, Philpott C, Schilder AGM, et al. Systemic and topical antibiotics for chronic rhinosinusitis. Cochrane Database Syst Rev. 2016 Apr 26;4(4):CD011994.

18. Thoma DS, Zeltner M, Hüsler J, Hämmerle CHF, Jung RE. EAO Supplement Working Group 4 - EAO CC 2015 Short implants versus sinus lifting with longer implants to restore the posterior maxilla: a systematic review. Clin Oral Implants Res. 2015 Sep;26 Suppl 11:154–69.

19. Rudmik L, Soler ZM. Medical Therapies for Adult Chronic Sinusitis: A Systematic Review. JAMA. 2015 Sep 1;314(9):926–39.

20. Atieh MA, Alsabeeha NH, Tawse-Smith A, Faggion CM, Duncan WJ. Piezoelectric surgery vs rotary instruments for lateral maxillary sinus floor elevation: a systematic review and meta-analysis of intra- and postoperative complications. Int J Oral Maxillofac Implants. 2015;30(6):1262–71.

21. Ahovuo-Saloranta A, Rautakorpi UM, Borisenko O V, Liira H, Williams JW, Mäkelä M. Antibiotics for acute maxillary sinusitis in adults. Cochrane Database Syst Rev. 2014 Feb 11;(2):CD000243.

22. Lemiengre MB, van Driel ML, Merenstein D, Young J, De Sutter AIM. Antibiotics for clinically diagnosed acute rhinosinusitis in adults. Cochrane Database Syst Rev. 2012 Oct 17;10:CD006089.

23. Guarch Ibáñez B, Buñuel Álvarez JC, López Bermejo A, Mayol Canals L. [The role of antibiotics in acute sinusitis: a systematic review and meta-analysis]. An Pediatr (Barc). 2011 Mar;74(3):154–60.

24. Falagas ME, Karageorgopoulos DE, Grammatikos AP, Matthaiou DK. Effectiveness and safety of short vs. long duration of antibiotic therapy for acute bacterial sinusitis: a meta-analysis of randomized trials. Br J Clin Pharmacol. 2009 Feb;67(2):161–71.

25. Young J, De Sutter A, Merenstein D, van Essen GA, Kaiser L, Varonen H, et al. Antibiotics for adults with clinically diagnosed acute rhinosinusitis: a meta-analysis of individual patient data. Lancet. 2008 Mar 15;371(9616):908–14.

26. Falagas ME, Giannopoulou KP, Vardakas KZ, Dimopoulos G, Karageorgopoulos DE. Comparison of antibiotics with placebo for treatment of acute sinusitis: a meta-analysis of randomised controlled trials. Lancet Infect Dis. 2008 Sep;8(9):543–52.

27. Karageorgopoulos DE, Giannopoulou KP, Grammatikos AP, Dimopoulos G, Falagas ME. Fluoroquinolones compared with beta-lactam antibiotics for the treatment of acute bacterial sinusitis: a meta-analysis of randomized controlled trials. CMAJ. 2008 Mar 25;178(7):845–54.

28. Ioannidis JP, Contopoulos-Ioannidis DG, Chew P, Lau J. Meta-analysis of randomized controlled trials on the comparative efficacy and safety of azithromycin against other antibiotics for upper respiratory tract infections. J Antimicrob Chemother. 2001 Nov;48(5):677–89.

29. Benninger MS, Sedory Holzer SE, Lau J. Diagnosis and treatment of uncomplicated acute bacterial rhinosinusitis: summary of the Agency for Health Care Policy and Research evidence-based report. Otolaryngol Head Neck Surg. 2000 Jan;122(1):1–7.

30. de Ferranti SD, Ioannidis JP, Lau J, Anninger W V, Barza M. Are amoxycillin and folate inhibitors as effective as other antibiotics for acute sinusitis? A meta-analysis. BMJ. 1998 Sep 5;317(7159):632–7.

31. de Bock GH, Dekker FW, Stolk J, Springer MP, Kievit J, van Houwelingen JC. Antimicrobial treatment in acute maxillary sinusitis: a meta-analysis. J Clin Epidemiol. 1997 Aug;50(8):881–90.

32. Grunau O, Terheyden H. Lateral augmentation of the sinus floor followed by regular implants versus short implants in the vertically deficient posterior maxilla: a systematic review and timewise meta-analysis of randomized studies. Int J Oral Maxillofac Surg. 2023 Jul;52(7):813–24.

33. Strietzel FP. [Sinus floor elevation and augmentation. Evidence-based analysis of prognosis and risk factors]. Mund Kiefer Gesichtschir. 2004 Mar;8(2):93–105.

34. Jamali S, Nasrabadi N, Payahoo S, Darvish M, Ahmadizadeh H, Khosravi S. Management of the maxillary sinus complications after dental implantation: a systematic review and meta-analysis. Braz Dent Sci. 2020 Mar 31;23(2).

35. Duttenhoefer F, Souren C, Menne D, Emmerich D, Schön R, Sauerbier S. Long-term survival of dental implants placed in the grafted maxillary sinus: systematic review and meta-analysis of treatment modalities. PLoS One. 2013;8(9):e75357.

**Table S3.** Summary of Certainty of Evidence (GRADE) for Key Outcomes Related to Antibiotic Use in SFE Procedures.

| Outcome | Study design | Risk of bias | Inconsistency | Indirectness | Imprecision | Other considerations | Participants (studies) | Effect estimate | Certainty (GRADE) |
| --- | --- | --- | --- | --- | --- | --- | --- | --- | --- |
| Antibiotics for infection prevention in SFE | Mostly observational; few RCTs | Moderate to high | Serious (heterogeneity in regimens) | No | Serious (small samples, wide CI) | — | >18,900 patients (7 SRs) | Infection rates 0.3% to 35% | ⬤⬤◯◯ Low |
| Antibiotics for infection management (graft/sinusitis) | Case series; observational | High | Serious | No | Very serious | — | ~3,000 patients (3 SRs) | Resolution >90% with combined therapy | ⬤◯◯◯ Very Low |
| Implant survival with vs. without antibiotic prophylaxis | Mixed (incl. RCTs and cohort) | Moderate | Not serious | No | Moderate | Large sample size | >28,000 implants | Survival >90% across studies | ⬤⬤⬤◯ Moderate |
| Comparison of different antibiotic regimens | Observational | Moderate | Serious | No | Serious | — | Varied; not consistently reported | No regimen clearly superior | ⬤⬤◯◯ Low |

Abbreviations: SFE = Sinus Floor Elevation; RCT = Randomized Controlled Trial; SR = Systematic Review; GRADE = Grading of Recommendations Assessment, Development and Evaluation. Certainty levels: ⬤⬤⬤⬤ High; ⬤⬤⬤◯ Moderate; ⬤⬤◯◯ Low; ⬤◯◯◯ Very Low.
